# Accounting for Imperfect Detection Is Critical for Inferring Marine Turtle Nesting Population Trends

**DOI:** 10.1371/journal.pone.0062326

**Published:** 2013-04-24

**Authors:** Joseph B. Pfaller, Karen A. Bjorndal, Milani Chaloupka, Kristina L. Williams, Michael G. Frick, Alan B. Bolten

**Affiliations:** 1 Caretta Research Project, Savannah, Georgia, United States of America; 2 Archie Carr Center for Sea Turtle Research and Department of Biology, University of Florida, Gainesville, Florida, United States of America; 3 Ecological Modeling Services P/L, University of Queensland, St. Lucia, Queensland, Australia; Hawaii Pacific University, United States of America

## Abstract

Assessments of population trends based on time-series counts of individuals are complicated by imperfect detection, which can lead to serious misinterpretations of data. Population trends of threatened marine turtles worldwide are usually based on counts of nests or nesting females. We analyze 39 years of nest-count, female-count, and capture-mark-recapture (CMR) data for nesting loggerhead turtles (*Caretta caretta*) on Wassaw Island, Georgia, USA. Annual counts of nests and females, not corrected for imperfect detection, yield significant, positive trends in abundance. However, multistate open robust design modeling of CMR data that accounts for changes in imperfect detection reveals that the annual abundance of nesting females has remained essentially constant over the 39-year period. The dichotomy could result from improvements in surveys or increased within-season nest-site fidelity in females, either of which would increase detection probability. For the first time in a marine turtle population, we compare results of population trend analyses that do and do not account for imperfect detection and demonstrate the potential for erroneous conclusions. Past assessments of marine turtle population trends based exclusively on count data should be interpreted with caution and re-evaluated when possible. These concerns apply equally to population assessments of all species with imperfect detection.

## Introduction

Many wildlife monitoring programs worldwide use time-series counts of animals to infer population trends [1], which are widely used for global biodiversity assessments [2], [3]. However, the interpretation of trends from raw count data is often complicated by imperfect detection, a phenomenon associated with double-counting, misidentifying, or overlooking individuals present during a sampling period [4]. Imperfect detection may bias inferences and lead to serious misinterpretations of data unless changes in detection probability are explicitly considered.

Capture-mark-recapture (CMR) methods applied in combination with multistate open robust-design (MSORD) statistical modeling approaches are designed to account for changes in detection probability of animals at a given sampling location within and between sampling periods [5]–[7]. This approach has provided reliable estimates of population abundance, detection probabilities, and key demographic parameters [8]–[10], and is essential for diagnosing causes of population trends and evaluating management effectiveness [8], [11].

Marine turtle populations, like those of other long-lived marine species, are inherently difficult to monitor, which increases their vulnerability to overexploitation [12], [13]. Long lifespans, delayed sexual maturity, and wide-ranging migrations prevent direct monitoring of individuals during most life stages, especially as juveniles. Assessments of marine turtle populations usually rely on data from nesting beaches; counts of females, nests, and/or tracks left by nesting females provide the primary metrics for evaluation of population trends [14]–[16]. The National Research Council [15] has stressed the limitations of assessments that rely exclusively on such abundance information for monitoring marine turtle population trends. Because many marine turtle populations are threatened, failing to accurately assess population trends may have critical effects on the long-term conservation and management of these populations.

We analyze 39 years (1973–2011) of nest-count, female-count, and CMR data for threatened loggerhead turtles (*Caretta caretta*) nesting on Wassaw Island, Georgia, USA. We compare trends in abundance based on nest-count and female-count data with trends based on estimates of female abundance corrected for imperfect detection using the MSORD statistical modeling approach. We demonstrate how integrating abundance data with statistically rigorous estimates of imperfect detection can affect interpretations of long-term trends in marine turtle nesting populations.

## Methods

### Statement of Ethics

Animal ethics clearance was obtained from Georgia Department of Natural Resources, Wildlife Resources Division (Scientific Collecting Permit no. 6502 & 29-WBH-12-99), permitting the staff of the Caretta Research Project to conduct nocturnal patrols, as well as tag and obtain standard data from live turtles. Under Georgia state law, the animal welfare ethics of all wildlife research is approved by the Scientific Collecting Permit evaluation committee. Federal approval for this research was granted under US Fish and Wildlife Service permit no. 41620-12-018.

### Capture-mark-recapture and Trend Analyses

During the loggerhead nesting season (May–August) from 1973 to 2011, nocturnal patrols were conducted on Wassaw Island, GA, USA (31.89°N 80.97°W). All loggerhead nests and females that were encountered were counted, and females were checked for and, if necessary, fitted with individualized tags (see Text S1 for patrolling and tagging details).

Open robust design capture-mark-recapture histories were compiled for 1164 nesting loggerheads tagged on Wassaw Island over the 39-year period. The primary sampling occasions consisted of 39 annual summer nesting seasons with eight 13-day secondary sampling occasions (3 May –22 August) within each primary occasion (312 sampling occasions). The 13-day secondary sampling period is the mean interval between successive clutches within a season for individual loggerheads in this population.

Based on careful consideration of the biological issues inherent to long-term studies of long-lived species [17]–[19] and the limitations associated with fitting complex, highly parameterized models to sparse datasets [8], [18], we used the multistate open robust design (MSORD) statistical modeling approach [18] in Program MARK [20] to fit models of different underlying structure while simultaneously estimating demographic parameters and population abundance. This integrated approach maintains the variance-covariance structure throughout model fitting and parameter estimation, which provides more robust estimates and avoids over parameterization [8]. The MSORD approach explicitly accounts for temporary emigration due to skipped breeding behavior and the presence of transient individuals (those seen only once), two biological phenomena inherent to sea turtle breeding behavior [7], [9], [17]. The two states in the multistate framework were nesters (observed state and available for capture) and the unobserved ‘non-nester’ state for those turtles that skipped nesting in a given season. It must be assumed that nester and non-nester states have the same survival probability, while arrival, departure and detection parameters for the non-nester state are fixed at zero since they are not available for capture [8]. We fitted models comprising time-constant and time-specific transition probabilities between nester and non-nester states, and, for the nester state, we fitted models comprising time-constant and time-specific intra- and inter-seasonal probabilities of survival, arrival, departure and detection. When full time dependence was detected, we performed ad hoc step-function analyses to identify blocks of years (epochs) with similar parameter estimates and fitted new epoch-specific models to reduce the number of parameters and improve precision of these parameter estimates. The parameter index matrices of the suite of models that were fitted in Program MARK in this study can be made available by the authors upon request.

The best-fit model based on the Akaike Information Criterion corrected for sample size and possible overdispersion (QAICc) was then used to estimate parameters such as apparent annual survival, conditional breeding (nesting), seasonal arrival and departure, and detection probabilities. Model goodness-of-fit was assessed using procedures in Prince & Chaloupka [9]. The MSORD approach in Program MARK was also used to estimate derived parameters such as annual female abundance and mean nesting season residency or ‘stopover’ time [6], [7], which is the basis for estimating annual clutch frequency, the mean number of clutches laid per female per year [7], [21]. Although the primary goal of this study was to estimate annual female abundance corrected for changes in detection, we estimated all demographic parameters simultaneously across all years to integrate across the variance-covariance structure of the best-fit model and provide robust estimates for other parameters to help diagnose patterns in long-term trends [9], [10].

Trends in nest counts, female counts, and estimates of female abundance corrected for imperfect detection were then estimated using generalized least squares with first-order autocorrelated error structure [22]. Higher-order autocorrelation was explored, but was unwarranted [22]. Statistical analyses were performed with S-Plus version 8.1 software (TIBCO Spotfire Software, Inc.) with α = 0.05.

## Results

From 1973 through 2011, 1164 female loggerheads were tagged on Wassaw Island, and 399 marked individuals were recaptured at least once. The most parsimonious MSORD model fitted to the 1164 CMR histories comprised time-specific inter-seasonal but constant intra-seasonal detection probabilities that differ within four distinct epochs (1973–1986, 1987–1993, 1994–2002, 2003–2011; see Table S1 for parameter estimates), and constant intra-seasonal probability of arriving and remaining in the study area (Table 1). There was strong support for this model (>93%) compared to the next best models (Table 1).

**Table 1:** Summary of model parameters for MSORD models 1–8.

model	description	QAICc	ΔQAICc	QAICc weights	deviance
1	*S*(TSM)*ψ*(.)*pent*(.t)*φ*(.TSM)*p*(.4,t)	10709.23	0.00	0.9345	10623.69
2	*S(*TSM)*ψ*(.)*pent*(.X10yr,t)*φ*(.TSM)*p*(.4,t)	10714.55	5.32	0.0654	10593.52
3	*S*(TSM)*ψ*(.t)*pent*(.t)*φ*(.TSM)*p*(.4,t)	10734.51	25.28	0.00	10579.60
4	*S*(TSM)*ψ*(.)*pent*(.X10yr,t)*φ*(.X10yr,TSM)*p*(.4,t)	10746.89	37.67	0.00	10602.63
5	*S*(TSM)*ψ*(.)*pent*(.t)*φ*(.TSM)*p*(t,t)	10750.29	41.06	0.00	10219.25
6	*S*(TSM)*ψ*(.)*pent*(.X10yr,t)*φ*(.X10yr,TSM)*p*(t,t)	10795.86	86.63	0.00	10225.05
7	*S*(TSM)*ψ*(.)*pent*(.t)*φ*(.t)*p*(.4,t)	10818.17	108.94	0.00	10730.56
8	*S*(.)*ψ*(.)*pent*(.t)*φ*(.TSM)*p*(.4,t)	10831.99	122.76	0.00	10748.53

Model descriptions follow Kendall and Bjorkland [7]. QAICc = sample size and overdispersion corrected Akaike Information Criterion based on *c*-hat = 1.037, ΔQAICc = difference between each model and the best-fit model 1, QAICc weights = weight of evidence in support of the model. Model parameters: *S*(TSM) = constant time-since-marking (TSM) annual survival probability; *S*(.) = constant annual survival probability; *ψ*(.) = constant transition probability from the nester to non-nester state and visa versa; *ψ*(.t) = constant transition probability from the nester to non-nester state, but time-specific transition probability from non-nester to nester state**;** *pent*(.t) = time-specific inter-seasonal but constant intra-seasonal probability of arrival; *pent*(.X10yr,t) = time-specific inter-seasonal but constant intra-seasonal arrival probability within 10-year epochs; *φ*(.TSM) = time-since-marking inter-seasonal but constant intra-seasonal probability of remaining; *φ*(.X10yr,TSM) = time-since-marking inter-seasonal but constant intra-seasonal probability of remaining within 10-year epochs; *φ*(.t) = constant intra-seasonal but time-specific inter-seasonal probability of remaining in the study area; p(.4,t) = time-specific inter-seasonal but constant intra-seasonal detection probabilities that differ within four epochs; and *p*(t,t) = time-specific intra-seasonal and inter-seasonal detection probabilities.

**Figure 1:**
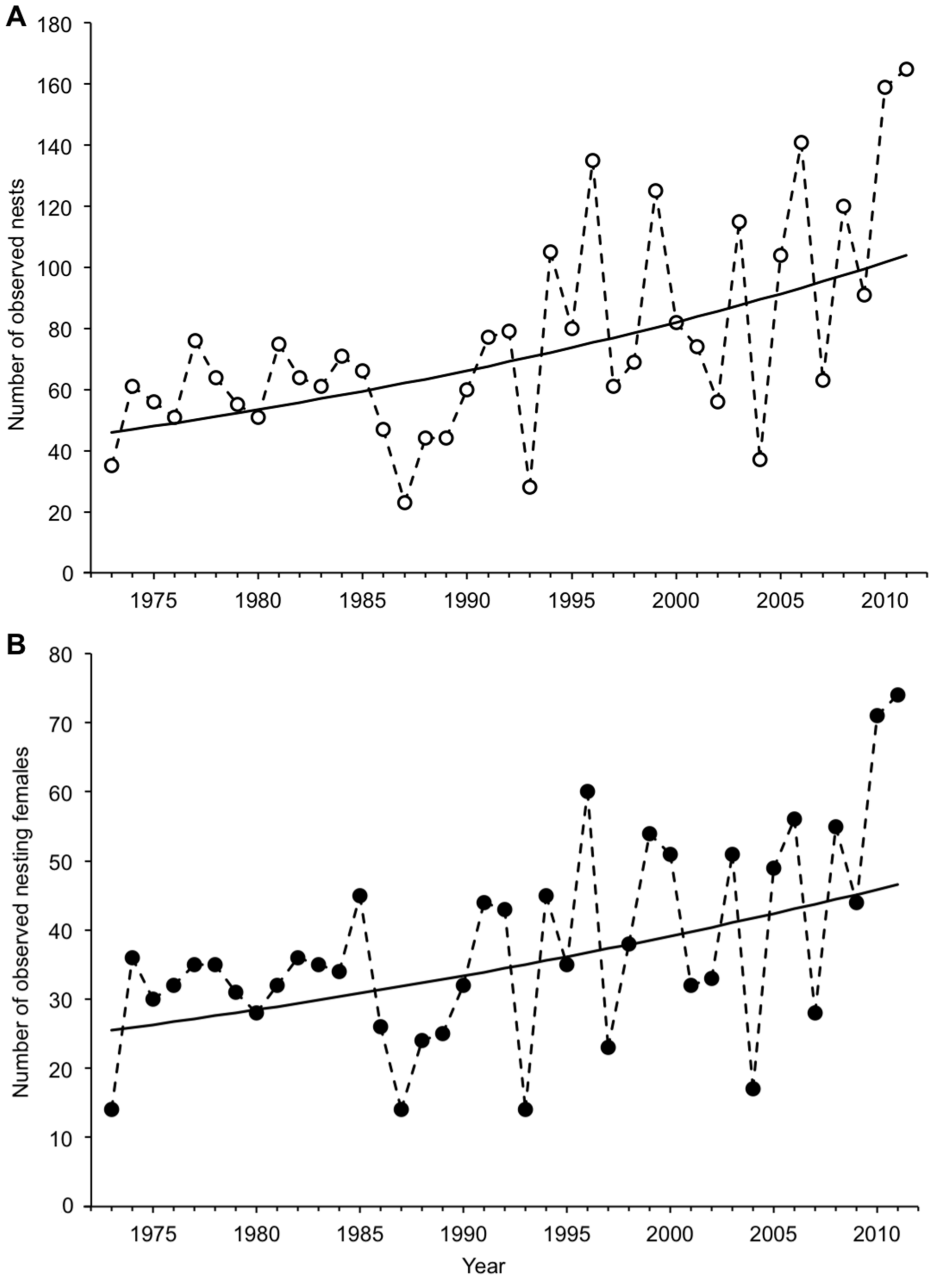
Annual number of observed loggerhead nests and nesting females on Wassaw Island. All (A) nests (open circles) and (B) nesting females (solid circles) that were encountered during nocturnal patrols were counted for 39 years (1973–2011) on Wassaw Island, GA, USA. Solid black lines show log-linear regressions using generalized least squares with first-order autocorrelated error structure. Both trends are significant (see text).

**Figure 2:**
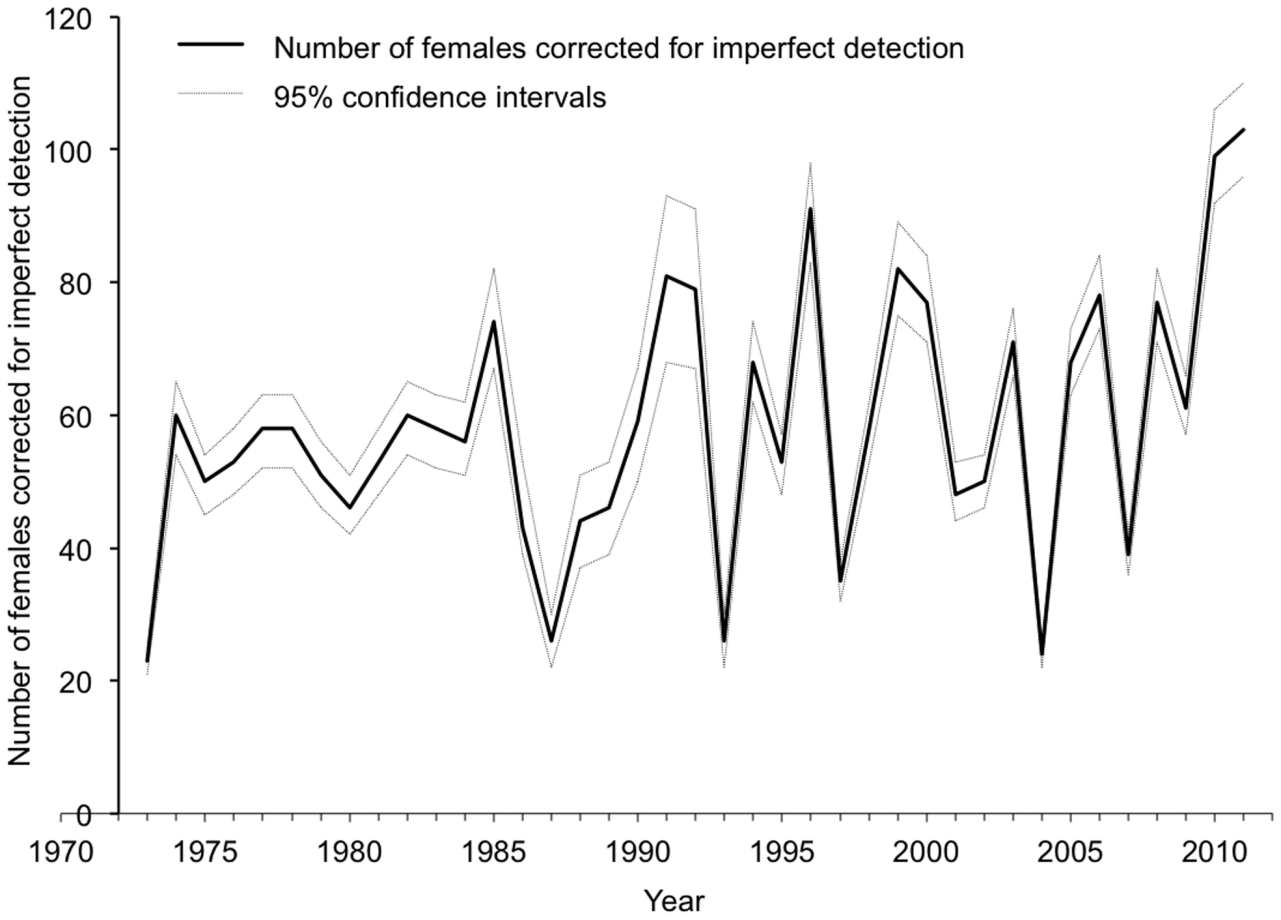
Annual number of nesting female loggerheads on Wassaw Island corrected for imperfect detection. Estimates (solid line) were derived from the best-fit MSORD model in Table 1. Dotted lines show 95% confidence intervals. No significant trend (see text).

During the study period, there were significant increases in annual nest counts (Fig. 1A and Table S2; generalized least squares, t_39_ = 3.83, *p*-value = 0.0005) and observed female counts (Fig. 1B and Table S2; generalized least squares, t_39_ = 3.10, *p*-value = 0.0037). The mean annual growth rates for these metrics were estimated to be 2.17% nests per year (95% CI = 1.05–3.30%) and 1.60% females per year (95% CI = 0.58–2.63%). However, the trend based on estimates of female abundance corrected for imperfect detection shows no significant change (Fig. 2 and Table S2; generalized least squares, t_39_ = 1.73, *p*-value = 0.0912). The number of females nesting each season has fluctuated around a long-term mean of ca. 60 individuals. A constant mean clutch frequency of 2.44 nests/female (95% CI = 2.26–2.62) during the study period was estimated from the best-fit MSORD model.

## Discussion

Nest-count and female-count data from a program that attempted to count every nest and tag every female for 39 years on Wassaw Island exhibit trends that suggest an increasing nesting population of loggerhead turtles. Annual population estimates corrected for imperfect detection of nesting females, however, demonstrate that this nesting population has remained at a relatively constant abundance over this period. These results demonstrate how integrating abundance data with statistically rigorous estimates of detection probability can strongly affect interpretations of long-term population trends if changes in detection probability are not explicitly considered. The long-term stability of this nesting subpopulation of loggerheads has been questioned in the past, and accurate assessments of population trends are critical for identifying the causes underlying these trends and to predict future trends.

Nest counts are not necessarily reliable indices of female abundance in marine turtle nesting populations. In addition to imperfect detection, trends in nest counts could result from changes in mean clutch frequency rather than changes in the numbers of females [23]. The apparent increase in nest counts in our study was not a result of increasing mean clutch frequency. Based on constant residency (stopover) time estimates from the best-fit MSORD model, mean clutch frequency remained constant during the study period. Therefore, because female abundance and clutch frequency did not change, the apparent increase in nest counts is likely due to increased nest detection, similar to that of female counts, and not to an increase in nest abundance.

Changes in detection may result from changes in population fidelity to a sampling location or from changes in methodology. Loggerheads nesting on Wassaw Island, as well as other marine turtle nesting populations, are known to show fidelity to a particular nesting beach while also using other nearby nesting beaches within and between breeding seasons [24], [25]. Changes in methodology that may have contributed to increased detection probabilities on Wassaw Island during the study period include switching from walking to driving patrols, and changing the type and number of tags applied to each turtle resulting in improved tag retention and individual identification. Changes in observer skill over time likely contribute to changes in detection as well [26], [27]. Even the tightest standardization in field protocols and observer skill levels may still fail to produce constant detection probability [4]. Thus, statistically rigorous estimates of detection probability should always be considered. This is especially important for those working with long-term datasets that include many years of data without sufficient metadata for current researchers to account for past changes in methodology.

Population trends of threatened and endangered marine turtles worldwide are usually based on counts of nests, nesting females, and/or tracks left by nesting females [15]. Status assessments [28], [29] and recovery plans [30] developed by government agencies and international conservation organizations are often based on such trend analyses. Our results demonstrate that abundance data that do not account for changes in detection may lead to erroneous conclusions. For some populations of marine turtles that are threatened, accurate interpretations of long-term population trends are critical for evaluating effective management actions. Past assessments of marine turtle population trends based exclusively on abundance data should be interpreted with caution and re-evaluated when possible.

Our results apply to a wide range of species – many of which are threatened or endangered – that are currently monitored with raw count data not corrected for imperfect detection. Understanding the consequences of failing to account for imperfect detection when inferring population trends is critical for developing effective wildlife monitoring programs and avoiding erroneous conclusions that may detrimentally affect already threatened and endangered populations.

## Supporting Information

Table S1 **Summary of detection probabilities estimated from best-fit MSORD model.**
(DOCX)

Table S2 **Annual counts of nests and nesting females on Wassaw Island, and annual estimates of nesting female abundance derived from the best-fit MSORD model.**
(DOCX)

Text S1 **Patrolling and tagging details for sea turtle research conducted on Wassaw Island.**
(DOCX)
