# Supplementary material for: Accounting for Imperfect Detection Is Critical for Inferring Marine Turtle Nesting Population Trends
Source: PLoS One. 2013 Apr 24;8(4):e62326. doi: 10.1371/journal.pone.0062326 (PMC3634727; doi:10.1371/journal.pone.0062326)
Supplement: Table S1 — Summary of detection probabilities estimated from best-fit MSORD model. (DOCX) [file pone.0062326.s001.docx]

**Table S1.** Summary of detection probabilities estimated from best-fit MSORD model.

| year block | within season sampling occasion | probability of detection (*p*) | lower 95% C.I. | upper 95% C.I. |
| --- | --- | --- | --- | --- |
| 1973–1986 | 1 | NE | NE | NE |
|  | 2 | 0.40 | 0.27 | 0.55 |
|  | 3 | 0.66 | 0.54 | 0.76 |
|  | 4 | 0.64 | 0.54 | 0.73 |
|  | 5 | 0.51 | 0.42 | 0.60 |
|  | 6 | 0.37 | 0.29 | 0.46 |
|  | 7 | 0.12 | 0.08 | 0.17 |
|  | 8 | NE | NE | NE |
| 1987–1993 | 1 | NE | NE | NE |
|  | 2 | 0.26 | 0.14 | 0.42 |
|  | 3 | 0.54 | 0.38 | 0.70 |
|  | 4 | 0.52 | 0.37 | 0.66 |
|  | 5 | 0.40 | 028 | 0.54 |
|  | 6 | 0.32 | 0.22 | 0.44 |
|  | 7 | 0.22 | 0.15 | 0.33 |
|  | 8 | NE | NE | NE |
| 1994–2002 | 1 | NE | NE | NE |
|  | 2 | 0.72 | 0.50 | 0.87 |
|  | 3 | 0.67 | 0.56 | 0.78 |
|  | 4 | 0.64 | 0.53 | 0.73 |
|  | 5 | 0.60 | 0.50 | 0.69 |
|  | 6 | 0.36 | 0.28 | 0.45 |
|  | 7 | 0.10 | 0.06 | 0.15 |
|  | 8 | NE | NE | NE |
| 2003–2011 | 1 | NE | NE | NE |
|  | 2 | 0.70 | 0.47 | 0.84 |
|  | 3 | 0.86 | 0.77 | 0.91 |
|  | 4 | 0.72 | 0.64 | 0.79 |
|  | 5 | 0.67 | 0.58 | 0.75 |
|  | 6 | 0.48 | 0.38 | 0.57 |
|  | 7 | 0.16 | 0.09 | 0.18 |
|  | 8 | NE | NE | NE |

NE = not estimated
