# Supplementary material for: Accounting for Imperfect Detection Is Critical for Inferring Marine Turtle Nesting Population Trends
Source: PLoS One. 2013 Apr 24;8(4):e62326. doi: 10.1371/journal.pone.0062326 (PMC3634727; doi:10.1371/journal.pone.0062326)
Supplement: Table S2 — Annual counts of nests and nesting females on Wassaw Island, and annual estimates of nesting female abundance derived from the best-fit MSORD model. (DOCX) [file pone.0062326.s002.docx]

**Table S2.** Annual counts of nests and nesting females on Wassaw Island, and annual estimates of nesting female abundance derived from the best-fit MSORD model.

| year | observed nests | observed females | estimated females | lower 95% C.I. | upper 95% C.I. |
| --- | --- | --- | --- | --- | --- |
| 1973 | 35 | 14 | 23 | 21 | 25 |
| 1974 | 61 | 36 | 60 | 54 | 65 |
| 1975 | 56 | 30 | 50 | 45 | 54 |
| 1976 | 51 | 32 | 53 | 48 | 58 |
| 1977 | 76 | 35 | 58 | 52 | 63 |
| 1978 | 64 | 35 | 58 | 52 | 63 |
| 1979 | 55 | 31 | 51 | 46 | 56 |
| 1980 | 51 | 28 | 46 | 42 | 51 |
| 1981 | 75 | 32 | 53 | 48 | 58 |
| 1982 | 64 | 36 | 60 | 54 | 65 |
| 1983 | 61 | 35 | 58 | 52 | 63 |
| 1984 | 71 | 34 | 56 | 51 | 62 |
| 1985 | 66 | 45 | 74 | 67 | 82 |
| 1986 | 47 | 26 | 43 | 39 | 53 |
| 1987 | 23 | 14 | 26 | 22 | 30 |
| 1988 | 44 | 24 | 44 | 37 | 51 |
| 1989 | 44 | 25 | 46 | 39 | 53 |
| 1990 | 60 | 32 | 59 | 50 | 67 |
| 1991 | 77 | 44 | 81 | 68 | 93 |
| 1992 | 79 | 43 | 79 | 67 | 91 |
| 1993 | 28 | 14 | 26 | 22 | 30 |
| 1994 | 105 | 45 | 68 | 62 | 74 |
| 1995 | 80 | 35 | 53 | 48 | 57 |
| 1996 | 135 | 60 | 91 | 83 | 98 |
| 1997 | 61 | 23 | 35 | 32 | 38 |
| 1998 | 69 | 38 | 58 | 53 | 62 |
| 1999 | 125 | 54 | 82 | 75 | 89 |
| 2000 | 82 | 51 | 77 | 71 | 84 |
| 2001 | 74 | 32 | 48 | 44 | 53 |
| 2002 | 56 | 33 | 50 | 46 | 54 |
| 2003 | 115 | 51 | 71 | 66 | 76 |
| 2004 | 37 | 17 | 24 | 22 | 25 |
| 2005 | 104 | 49 | 68 | 63 | 73 |
| 2006 | 141 | 56 | 78 | 73 | 84 |
| 2007 | 63 | 28 | 39 | 36 | 42 |
| 2008 | 120 | 55 | 77 | 71 | 82 |
| 2009 | 91 | 44 | 61 | 57 | 66 |
| 2010 | 159 | 71 | 99 | 92 | 106 |
| 2011 | 165 | 74 | 103 | 96 | 110 |
