# Supplementary material for: Accounting for Imperfect Detection Is Critical for Inferring Marine Turtle Nesting Population Trends
Source: PLoS One. 2013 Apr 24;8(4):e62326. doi: 10.1371/journal.pone.0062326 (PMC3634727; doi:10.1371/journal.pone.0062326)
Supplement: Text S1 — Patrolling and tagging details for sea turtle research conducted on Wassaw Island. (DOCX) [file pone.0062326.s003.docx]

**Text S1.** Patrolling and tagging details for sea turtle research conducted on Wassaw Island.

*Nocturnal patrols*

- Nocturnal patrols were conducted from ~2100 h to ~0600 h. Two groups of up to four people patrolled the beach using three- or four-wheel vehicles and/or by walking depending on the tide stage, weather conditions, and vehicle performance [1].

*Tags and tagging*

- External tags [1]: Turtles were single-tagged with monel metal tags (type 49, National Band and Tag Company, Newport, Kentucky, USA) from 1973–1974. Turtles were double-tagged with nylon jumbo Rototags (Dalton Supplies Ltd., Henley, England) and monel metal tags from 1975–1977. Turtles were double- or triple-tagged with plastic Riese tags (size 2, Dalton Supplies Ltd., Henley, England), nylon jumbo Rototags and monel metal tags from 1978–1986. Turtles were double-tagged with Inconel metal tags (style 681, National Band and Tag Company, Newport, Kentucky, USA) from 1987–2011.
- External tagging [1]: Single tagged turtles were tagged in either the first or second large scale on the posterior edge of the right front flipper. Double-tagged turtles were tagged in the first or second large scale on the posterior edge of both front flippers. Triple-tagged turtles were tagged in both front flippers and in the first or second large scale on the posterior edge of either the right or left rear flipper.
- Passive Integrated Transponder (PIT) tags and scanners [1]: Trovan brand (Douglas, UK) PIT tags and scanners were used from 1992–1998. Destron Fearing brand (South St. Paul, Minnesota, USA) PIT tags and scanners were used from 1999–2011. Destron Fearing brand PIT tag scanners can read Trovan brand PIT tags.
- PIT tagging [1]: PIT tags were implanted subcutaneously just proximal to the elbow region of the right front flipper.

*References*

1. Williams KL, Frick MG (2001) Results from the long-term monitoring of nesting loggerhead sea turtles (*Caretta caretta*) on Wassaw Island, Georgia: 1973–2000. NOAA Tech Memo NMFS-SEFSC-446. Available from: www.sefsc.noaa.gov.
